# Accumulation of 3‐aminopropylphosphonate in the ex vivo brain observed by phosphorus‐31 nuclear magnetic resonance

**DOI:** 10.1002/nbm.4721

**Published:** 2022-03-13

**Authors:** David Shaul, Benjamin Grieb, Naama Lev‐Cohain, Jacob Sosna, J. Moshe Gomori, Rachel Katz‐Brull

**Affiliations:** ^1^ Department of Radiology, Hadassah Medical Center Hebrew University of Jerusalem, The Faculty of Medicine Jerusalem Israel; ^2^ The Wohl Institute for Translational Medicine Jerusalem Israel; ^3^ Department of Psychiatry and Psychotherapie I (Weissenau), ZfP Suedwuerttemberg Ulm University Ravensburg Germany

**Keywords:** 3‐APP 3‐aminopropylphosphonate, ATP adenosine triphosphate, 3‐APA 3‐aminopropanephosphinic acid, brain, 31P phosphorous NMR spectroscopy

## Abstract

3‐aminopropylphosphonate (3‐APP) is known for its use as an exogenous indicator of extracellular volume and pH in phosphorus‐31 nuclear magnetic resonance (^31^P NMR) studies. We used 3‐APP for estimating the extracellular volume in NMR studies of several ex vivo preparations including retrograde perfused mouse heart (n = 4), mouse liver slices (n = 2), xenograft breast cancer tumors (n = 7, MCF7), and rat brain slices (n = 4). In the former three preparations, the 3‐APP signal was stable in lineshape and intensity for hours and the chemical shift of the signal in the presence of the biological sample was the same as in the perfusion medium without the biological sample. However, in studies of brain slices, the 3‐APP signal appeared split into two, with an upfield component (0.7 ± 0.1 ppm to the left) increasing with time and showing a wider linewidth (66.7 ± 12.6 vs. 39.1 ± 7.6 Hz, the latter is of the perfusion medium signal). This finding suggests that 3‐APP inadvertently accumulated in brain slices, most likely as a membrane bound form. This observation limits the use of 3‐APP as an inert biochemical indicator in brain preparations and should be taken into account when using 3‐APP in vivo.

## Graphical abstract

3‐Aminopropylphosphonate (3‐APP) is an exogenous indicator of extracellular volume and pH in ^31^P NMR studies. In perfused rat brain slices, the 3‐APP signal appeared split into two, with an upfield component (0.7 ± 0.1 ppm) increasing with time and showing a wider linewidth compared with the signal from the perfusion medium (66.7 ± 12.6 vs. 39.1 ± 7.6 Hz, respectively; *n* = 4). This finding suggests that 3‐APP accumulated in brain slices, most likely as a membrane bound form.

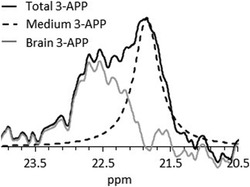

**Abbreviations used**
3‐APA: 3‐aminopropanephosphinic acid
3‐APP: 3‐aminopropylphosphonate
ATP: adenosine triphosphate

## 1 INTRODUCTION

3‐aminopropylphosphonate (3‐APP) is an exogenous marker, used predominantly to measure the extracellular pH of tumors in preclinical NMR studies.^1^, ^2^, ^3^ In 1994, it was shown to be nontoxic and membrane impermeant.^2^ Since then, it has been used by numerous groups to measure extracellular pH and to estimate extracellular volume in a variety of animal and tumor models.^1^, ^2^, ^3^

We were interested in using 3‐APP to characterize extracellular volume in a perfused tissue slices preparation and a perfused mouse heart model that we have been developing for monitoring hyperpolarized substrates metabolism (^13^C‐NMR) in tandem with phosphorus‐31 nuclear magnetic resonance (^31^P‐NMR) monitoring of pH and high energy phosphate status.^4^, ^5^ In such ^31^P‐NMR studies, the Pi signal is composed of both the extracellular Pi from the medium perfusing the slices (or heart) and the intracellular Pi within the cells in the slices (or heart). For this reason, it is difficult to estimate the extracellular volume from the Pi signal and there are no other intrinsic markers for the extracellular volume. 3‐APP, being membrane impermeant and having a distinguishable chemical shift at a higher field, appeared potentially useful.

We show here that while 3‐APP is useful for determining extracellular space in tissue slices of mice tumors and livers, and in perfused mouse hearts, it is not useful for this purpose in perfused rat cerebrum slices. In the latter, 3‐APP accumulated in the slices and for this reason could not serve as a biochemically inert indicator.

## 2 METHODS

### 2.1 Materials

3‐APP was purchased from Sigma‐Aldrich (Rehovot, Israel) and was added to the perfusion media of ex vivo preparations at a concentration of 1 mM (heart and brain) and 2 mM (MCF7 breast cancer tumors and liver).

### 2.2 Perfused ex vivo preparations

Tissue slices and organs were prepared and perfused in the NMR spectrometer as previously described for mouse liver^6^, ^7^ (n = 2), breast cancer xenograft tumors^8^ (n = 7), mouse heart^9^ (n = 4), and rat cerebrum^10^, ^11^, ^12^ (n = 4).

### 2.3 Ethics

The joint ethics committee (Institutional Animal Care and Use Committee) of the Hebrew University and Hadassah Medical Center approved the study protocols for animal welfare (protocol numbers: MD‐16‐14739‐1 and MD‐19‐15882‐3 [brain]; MD‐17‐15045‐5 [breast cancer tumors]; MD‐16‐14741‐1 [liver]; and MD‐16‐14724‐1 and MD‐19‐15827‐1 [heart]). The Hebrew University is an AAALAC International accredited institute. Care was taken to minimize pain and discomfort to the animals.

### 2.4 NMR spectroscopy

^31^P spectroscopy was performed in a 5.8‐T NMR spectrometer equipped with a broadband NMR probe (RS2D, Mundolsheim, France). The tissue was placed inside a 10‐mm NMR tube and connected to the perfusion system as previously described.^6^, ^7^, ^8^, ^9^, ^10^, ^11^, ^12^ The spectra were acquired using a nutation angle of 50° and a repetition time of 1.1 s. Each spectrum was obtained using 820 scans (total scan time of 15 min).

### 2.5 Spectral analysis

Spectral analysis was performed using MNova (Mestrelab Research, Santiago de Compostela, Spain). Each spectrum was zero‐filled to 16,384 points, drift‐corrected, exponentially multiplied with a 10‐Hz line broadening (LB), and baseline corrected. Calculation of the full width at half maximum (FWHM) of the 3‐APP signal in the brain slices was performed using the processed spectra ( ${\text{FWHM}}_{\text{processed}}$, with 10‐Hz LB) because the natural FWHM ( ${\text{FWHM}}_{\text{natural}}$) was difficult to assess due to the high noise level in the non line‐broadened spectra.

The ${\text{FWHM}}_{\text{natural}}$ of these signals was then derived using Equation (1).

(1) $${\text{FWHM}}_{\text{natural}}={\text{FWHM}}_{\text{processed}}-\mathrm{LB}.$$

We note that because 3‐APP accumulation in the brain affected the signal shape and the corresponding linewidth, improvement of signal‐to‐nosie ratio (SNR) by increasing the number of scans would not necessarily provide a better measure of the linewidth.

### 2.6 Calculation of 3‐APP accumulation in the brain

Deconvolution of the signals of 3‐APP in the perfusion medium (3‐APP_m_) and in the brain (3‐APP_b_) was performed in Excel (Microsoft, Ra′anana, Israel). The chemical shift of the 3‐APP_m_ signal was the same as its chemical shift in medium that did not contain the slices, and the chemical shift of 3‐APP_b_ was 0.7 ± 0.1 ppm upfield (n = 4). In the spectra recorded from the perfused brain slices, a Lorentzian lineshape was centered at the frequency of the 3‐APP_m_ signal and fitted to its linewidth (39.1 ± 7.6 Hz, n = 4). This fitted signal was then subtracted from the total 3‐APP signal (3‐APP_t_) to yield the 3‐APP_b_ signal. Then the integrated intensities of both components were normalized to the integrated intensity of the 3‐APP signal recorded from the medium alone (without the brain slices). Because the T_1_ of the 3‐APP_b_ signal is unknown, absolute quantification could not be performed.

## 3 RESULTS

**FIGURE 1:**
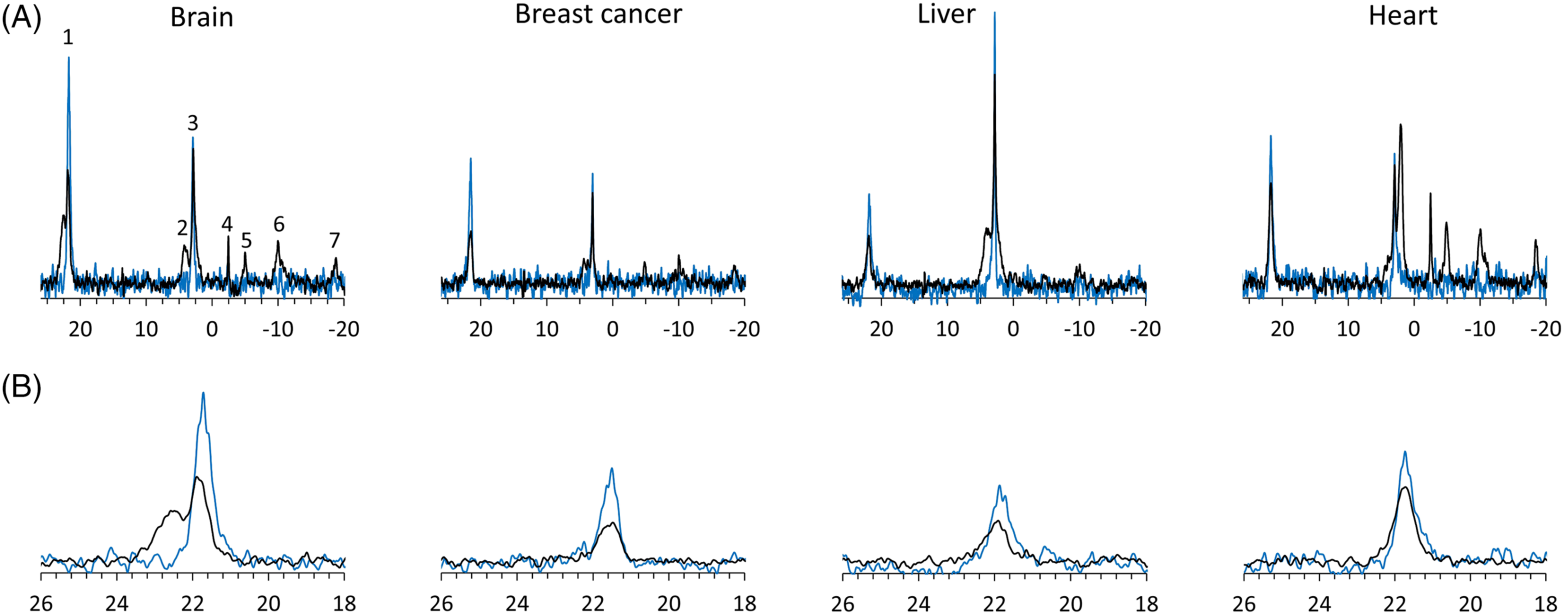
Phosphorus‐31 nuclear magnetic resonance (^31^P‐NMR) spectra acquired from ex vivo preparations of perfused brain slices, breast cancer slices, liver slices, and retrograde perfused heart. (A) Blue: spectra of the perfusion media for the various tissues supplemented with 3‐aminopropylphosphonate (3‐APP) (without the tissue). Black: spectra of the viable tissues perfused with these media. (B) Expansion of the 3‐APP signal of the spectra shown in (A). Only the spectrum of the brain slices shows an additional signal, upfield to the 3‐APP signal of the perfusion medium. Each spectrum was recorded using a flip angle of 50°, TR of 1.1 s, and 5740 averages (total scan time of 105 min). Signal assignment: 1, 3‐APP; 2, phosphomonoesters, 3, inorganic phosphate; 4, phosphocreatine; 5, γ‐adenosine triphosphate (γ‐ATP); 6, α‐ATP; 7, β‐ATP

Ex vivo preparations of brain slices, breast cancer slices (MCF7), liver slices, and a retrograde perfused mouse heart were perfused with media supplemented with 3‐APP, as described in the Methods section. ^31^P NMR spectra of the tissues, recorded under this condition, are shown in Figure 1A (black). The ^31^P spectra of the perfusion media without the involved tissue are shown in Figure 1A (blue). Figure 1B shows the expanded 3‐APP signal for each tissue. In the breast cancer, liver, and heart preparations, the 3‐APP signal of the medium alone was greater than the 3‐APP signal from the sample that contained the tissues. The difference between these two signals is proportional to the volume that is occupied by the tissue and, by inference, to the proportion of the intracellular volume and medium/extracellular volume detected by the NMR probe.^1^ However, in the brain, an additional wider signal (66.7 ± 12.6 Hz, n = 4) was observed at a higher field (0.7 ± 0.1 ppm to the left, n = 4) relative to the 3‐APP signal of the medium.

**FIGURE 2:**
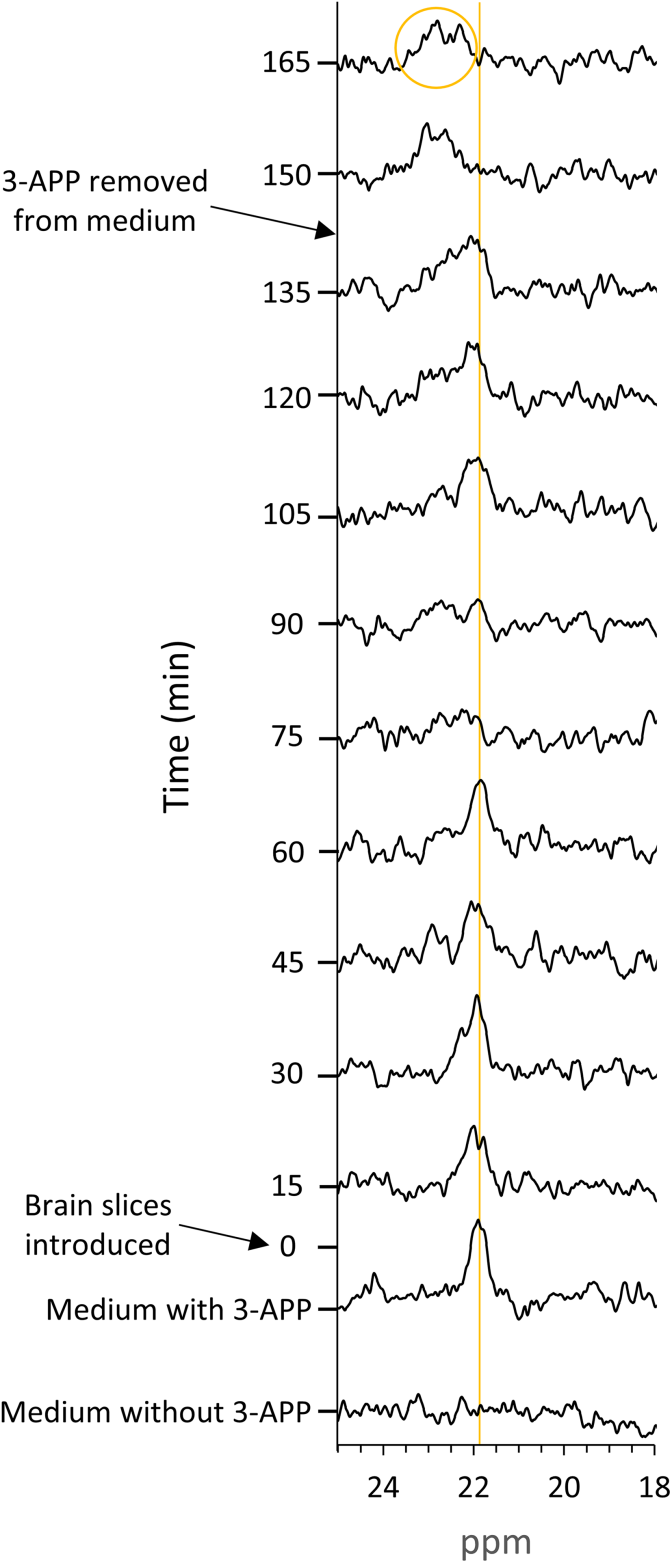
Demonstration of 3‐aminopropylphosphonate (3‐APP) accumulation in the rat brain slices. Representative ^31^P spectra of 3‐APP signal over time in the course of a wash‐in/wash‐out experiment. From the bottom up: the medium only (without brain slices) devoid of 3‐APP; supplementation of 3‐APP to the same medium; time 0 is the time at which the brain slices were introduced to the NMR tube; after 135 min, 3‐APP was washed out of the perfusion system by replacing the perfusion medium with a medium that did not contain 3‐APP. The yellow straight line indicates the position (in ppm) of the 3‐APP_m_ signal; the yellow circle highlights the 3‐APP_b_ signal (bound to the brain slices). Each spectrum was recorded using a flip angle of 50°, TR of 1.1 s, and 820 averages (total scan time of 15 min)

Figure 2 shows the dynamic nature of the 3‐APP signal from perfused rat brain slices. When the perfusion medium was supplemented with 3‐APP, a gradual increase in the 3‐APP_b_ signal was observed. When 3‐APP was washed out of the perfusion medium, the 3‐APP_b_ signal remained (Figure 2, yellow circle), while the 3‐APP_m_ signal disappeared as expected.

We note that the upfield chemical shift of the 3‐APP_b_ signal is in line with the acidity of the brain tissue in this preparation (approximately 6.9^1^), as the 3‐APP chemical shift is known to be higher at a lower pH.^2^

**FIGURE 3:**
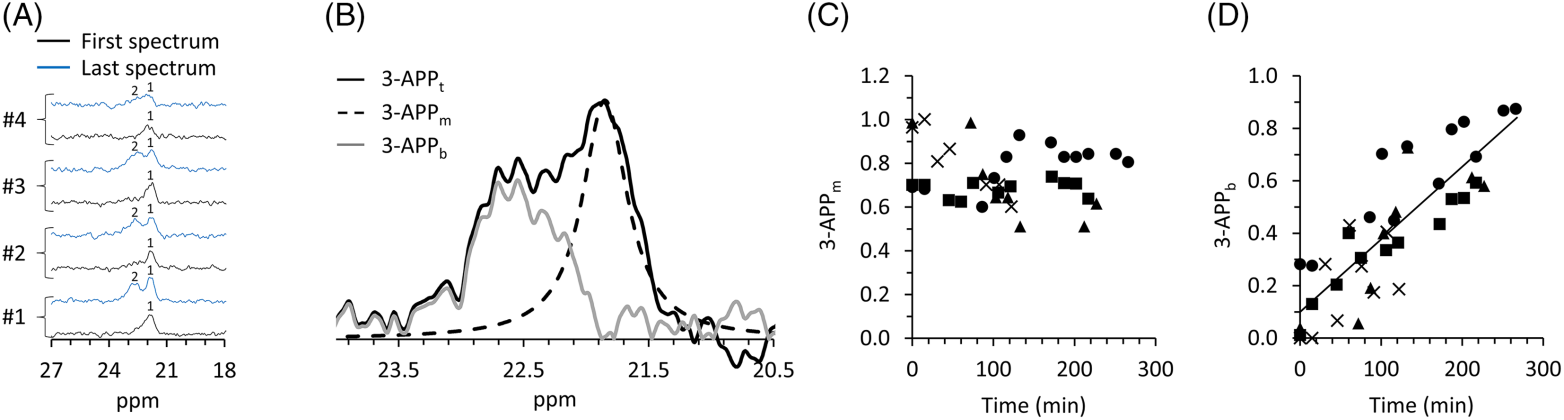
Accumulation of 3‐aminopropylphosphonate (3‐APP) in the brain. (A) Accumulation of 3‐APP in brain slices from four different rats. For each animal (#1 through 4), the spectrum in black shows the 3‐APP_t_ signal at the beginning of the incubation of the slices with 3‐APP, and the signal in blue shows the 3‐APP_t_ at the end of the incubation period. The 3‐APP_m_ and 3‐APP_b_ components of the 3‐APP_t_ signal are denoted as 1 and 2, respectively. The spectra were acquired with 820 scans, within 15 min each. (B) A typical ^31^P signal of 3‐APP_t_ acquired from rat brain slices perfused with a medium containing 3‐APP (1 mM). The acquired signal is shown in solid black; a Lorentzian lineshape fitting to the 3‐APP_m_ signal is shown with a dashed line; the result of subtracting 3‐APP_m_ signal from the 3‐APP_t_ signal is shown in gray and comprises the 3‐APPb signal. The spectrum was recorded using a flip angle of 50°, a TR of 1.1 s, and 820 averages (total scan time of 15 min). (C and D) Changes in the 3‐APP_m_ and the 3‐APP_b_ signals, respectively, with perfusion time. The levels of the 3‐APP_m_ and 3‐APP_b_ signals were normalized to the 3‐APP signal of the medium alone on each experimental time course (n = 4, each experimental time course is marked with a different marker type) recorded at the beginning of each experiment. The trend line in D demonstrates the increase with time of the 3‐APP_b_ signal (R^2^ = 0.7)

Figure 3A demonstrates the accumulation of 3‐APP in brain slices from four different rats (four different experimental days). The part of the ^31^P spectrum showing this signal is enlarged. The time of 3‐APP incubation was 3.2, 4.2, 3.5, and 2.25 h for the slices from animals #1, #2, #3, and #4, respectively (Figure 3A).

Figure 3B shows a typical 3‐APP signal deconvolution into its 3‐APP_m_ and 3‐APP_b_ signal components (see also the Methods section). In Figure 3C,D we show the change in these two components over time, in four different brain slice preparations (n = 4). It appears that while the 3‐APP_m_ signal remains unchanged over time (Figure 3C), the 3‐APP_b_ signal increases (Figure 3D). During the experimental time frame of about 4.5 h, the accumulation rate did not reach a plateau.

The pH values of the environments in which the 3‐APP_m_ and the Pi signals were present were calculated using the chemical shifts of both signals in the four experiments that were carried out on brain slices. The calculation was performed according to previously described equations.^1^, ^2^ The results are shown in Table 1, and suggest that the two pH determinations are congruent. A slightly lower pH value resulted from the calculation of pH that was based on the chemical shift of the 3‐APP_m_ signal. However, considering the small number of experiments and the SNR of the ^31^P spectra, we hesitate to draw further conclusions from this difference. Therefore, this pH comparison suggested that the two signals represent compounds that are found in the same environment. Based on the medium composition and the signal shape, the Pi signal appears to be predominantly from the perfusion medium. Therefore, it strongly suggests that the 3‐APP_m_ signal is found in the perfusion medium as well, as also suggested by the wash‐in/wash‐out experiments (Figure 2).

**TABLE 1:** Calculated environment pH based on the chemical shifts of the 3‐APP_m_ and Pi signals

Animal number	pH based on the chemical shift of 3‐APP_m_	pH based on the chemical shift of Pi
1	7.39	7.46
2	7.44	7.50
3	7.39	7.43
4	7.28	7.35
Average ± standard deviation	7.38 ± 0.06*	7.44 ± 0.06*

* Significance was calculated using a two‐tailed, paired, Student's *t*‐test (*p* = 0.003).

## 4 DISCUSSION

Multiple studies have established the use of 3‐APP as an indicator of extracellular volume and pH.^1^, ^2^, ^3^ In our work, we aimed at using 3‐APP to estimate the volume occupied by the tissue cells that is visible to the NMR probe. However, in the perfused rat brain slices preparation, the 3‐APP signal accumulated with time and displayed changes in the signal chemical shift and lineshape. This accumulation did not reach a plateau after 4.5 h of perfusion (Figure 3D). Because 3‐APP was not accumulating in the other tissues that we have studied (Figure 1), it is not likely that the accumulation in the perfused brain slices is due to a physical or other barrier that would lead to an increase in the concentration of this compound within the NMR probe. Therefore, we conclude that 3‐APP has been accumulating in the brain slices. In addition, the 3‐APP_b_ signal remained constant after 3‐APP was removed from the perfusion medium, reinforcing this conclusion (Figure 2). Unfortunately, we could not perform absolute quantification of the rate of 3‐APP accumulation in the brain. Bhujwalla et al.^1^ reported a longer T_1_ relaxation for 3‐APP in RIF‐1 tumors (6.1 s) compared with that of 3‐APP in saline (1.7 s). In our experimental setting, evaluation of 3‐APP T_1_ was not possible because the 3‐APP_b_ signal (and likely the content of 3‐APP in the brain slices) had increased with time.

The increase in the linewidth of 3‐APP_b_ compared with 3‐APP_m_ suggests that 3‐APP_b_ is membrane bound and not taken up into the intracellular space. This is because NMR linewidths increase with an increase in the molecular weight due to reduced molecular mobility.^13^ The NMR signals of membrane‐bound small molecules show the same effect, as they too have longer average rotational correlation times.

We note that the ^31^P signals of phosphodiesters were not observed in the rat brain slices. Such signals have been observed in the intact rat^14^, ^15^ and human^16^, ^17^, ^18^, ^19^ brain. However, the absence of phosphodiesters’ signals is consistent with earlier studies of rat brain slices.^20^, ^21^ This difference between the in vivo and ex vivo spectra is, to the best of our knowledge, as of yet unexplained.

Previous studies have shown that 3‐aminopropanephosphinic acid (3‐APA), which is the acidic form of 3‐APP, acts as a central and a peripheral GABA_B_ receptor agonist.^22^ This binding likely provides an explanation for 3‐APP accumulation in the brain. There are two implications of this observation: (1) The accumulation of 3‐APP in the brain impairs the ability to correctly evaluate the extracellular volume, as opposed to other tissues that we have experimented with; and (2) GABA_B_ agonists modulate brain activity and affect cerebral blood flow,^23^, ^24^, ^25^ thus 3‐APP may influence these factors as well, and this should be carefully considered when using 3‐APP in vivo.

In another aspect, this observation may open a window for new uses of 3‐APP in ^31^P NMR studies of the brain. This accumulation in the brain may be an indicator of pathologies and damaged tissues such as, for example, under stroke. 3‐APP may also become a marker of brain hypoxia and cell death due to the sensitivity of its chemical shift to tissue acidity.

In summary, this study demonstrates accumulation of 3‐APP in ex vivo perfused rat brain slices, as opposed to other ex vivo perfused tissue preparations (heart, liver, breast cancer tumor). This observation warrants caution as regards its ability to serve as an inert indicator on NMR studies.

## Data Availability

The data that support the findings of this study are available from the corresponding author upon reasonable request.
